# Vertical Electric-Field-Induced Switching from Strong to Asymmetric Strong–Weak Confinement in GaAs Cone-Shell Quantum Dots Using Transparent Al-Doped ZnO Gates

**DOI:** 10.3390/nano14211712

**Published:** 2024-10-27

**Authors:** Ahmed Alshaikh, Jun Peng, Robert Zierold, Robert H. Blick, Christian Heyn

**Affiliations:** Center for Hybrid Nanostructures (CHyN), University of Hamburg, Luruper Chaussee 149, 22761 Hamburg, Germany; ahmed.alshaikh@uni-hamburg.de (A.A.); jpeng@physnet.uni-hamburg.de (J.P.); rzierold@physnet.uni-hamburg.de (R.Z.); rblick@physnet.uni-hamburg.de (R.H.B.)

**Keywords:** Al-doped ZnO, GaAs quantum dot, photoluminescence, exciton, lifetime, Stark shift, strong and weak confinement

## Abstract

The first part of this work evaluates Al-doped ZnO (AZO) as an optically transparent top-gate material for studies on semiconductor quantum dots. In comparison with conventional Ti gates, samples with AZO gates demonstrate a more than three times higher intensity in the quantum dot emission under comparable excitation conditions. On the other hand, charges inside a process-induced oxide layer at the interface to the semiconductor cause artifacts at gate voltages above U≈ 1 V. The second part describes an optical and simulation study of a vertical electric-field (*F*)-induced switching from a strong to an asymmetric strong–weak confinement in GaAs cone-shell quantum dots (CSQDs), where the charge carrier probability densities are localized on the surface of a cone. These experiments are performed at low *U* and show no indications of an influence of interface charges. For a large *F*, the measured radiative lifetimes are substantially shorter compared with simulation results. We attribute this discrepancy to an *F*-induced transformation of the shape of the hole probability density. In detail, an increasing *F* pushes the hole into the wing part of a CSQD, where it forms a quantum ring. Accordingly, the confinement of the hole is changed from strong, which is assumed in the simulations, to weak, where the local radius is larger than the bulk exciton Bohr radius. In contrast to the hole, an increasing *F* pushes the electron into the CSQD tip, where it remains in a strong confinement. This means the radiative lifetime for large *F* is given by an asymmetric confinement with a strongly confined electron and a hole in a weak confinement. To our knowledge, this asymmetric strong–weak confinement represents a novel kind of quantum mechanical confinement and has not been observed so far. Furthermore, the observed weak confinement for the hole represents a confirmation of the theoretically predicted transformation of the hole probability density from a quantum dot into a quantum ring. For such quantum rings, application as storage for photo-excited charge carriers is predicted, which can be interesting for future quantum photonic integrated circuits.

## 1. Introduction

Following Fermi’s golden rule, the radiative lifetime of a semiconductor quantum dot (QD) is controlled by the overlap integral of the electron and hole wave functions [1]. This approach is usually assumed when the QD size is smaller than the bulk exciton Bohr radius $\lambda_{B}$ [1,2,3,4,5]. Often, QDs in a homogeneous medium are studied where the minimal lifetime is assumed for a maximum possible overlap integral of one. However, there are experimental data that demonstrate much shorter lifetimes. One scenario is the Purcell enhancement, where a QD is embedded in a photonic environment, which increases the photonic density of states [6,7]. The integration of QDs into such devices is often used to increase the intensity of the QD optical emission and the speed of the optical switching [8,9]. Another scenario is the weak confinement regime, where the QD size is larger than the bulk exciton Bohr radius  $\lambda_{B}$ [4,10,11]. Here, the charge carriers inside the QD can oscillate and collect a substantially higher recombination probability compared with the usual case of a strong confinement with a QD size smaller than $\lambda_{B}$. In this regime, the short radiative lifetimes are related to a giant oscillator strength [12,13].

The present work studies the radiative lifetime of GaAs cone-shell quantum dots (CSQDs) in a homogeneous medium experimentally and by means of simulations. CSQDs have a unique shape, where the charge carrier probability densities are localized on the surface of a cone. A vertical electric field *F* allows to shift the charge carriers over this cone shell, and simulations predict the *F*-induced transition of the hole probability density from a dot into a ring shape [14]. Since the radius of the confinement for the ring exceeds $\lambda_{B}$, one can expect an *F*-induced transition from a strong to weak confinement.

We see two major aspects of the present study. The central observation is the substantial discrepancy between the measured and simulated lifetimes at high vertical electric fields. This is explained by a transition from the usual strong into an asymmetric strong–weak confinement. To our knowledge, this represents a novel kind of quantum mechanical confinement and has not been observed so far. As a second aspect, the observed weak confinement for the hole represents a confirmation of the theoretically predicted [14] transformation of the hole probability density from a quantum dot into a quantum ring. For such quantum rings, application as storage for photo-excited charge carriers is predicted, which can be interesting for future quantum photonic integrated circuits [15].

## 2. Methods

The studied GaAs CSQD are fabricated in a self-assembled fashion during molecular beam epitaxy (MBE) by filling droplet-etched nanoholes in AlGaAs with a planar GaAs layer [14,16,17]. The shape of the bottom part of the CSQDs is given by the shape of the initial nanohole, which can be controlled by the LDE process conditions. The shape of the top part is formed by capillary during nanohole filling and is only poorly controlled so far. In a first approach, we studied the QD shape and size using AFM linescans [17]. However, since the linescans are taken from different samples, the QD height cannot be precisely determined from these data. The most accurate shape determination is based on a combination of AFM data of the nanohole template with PL measurements and simulations under a vertical electric field [14]. The resulting combination of a cone-like bottom part and a top part with a cone-like indentation yields a QD shape like the shell of a cone.

**Figure 1:**
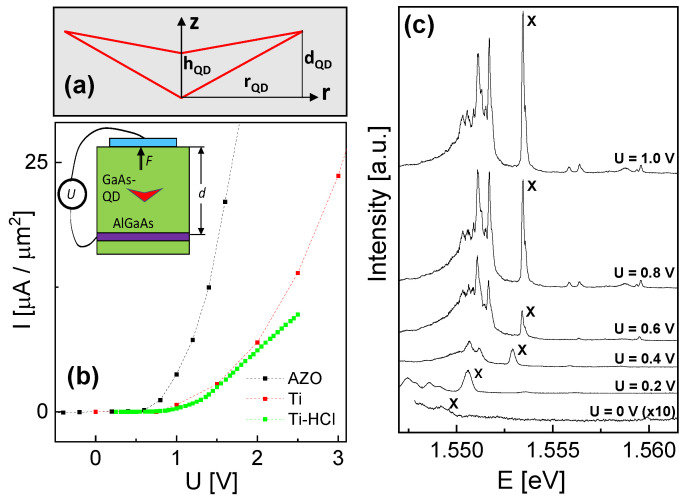
(**a**) Cross-sectional scheme of a rotationally symmetric CSQD with characteristic lengths $r_{QD}$, $d_{QD}$, and $h_{QD}$. (**b**) Measured current density between top and back gate as function of the gate voltage *U*. The respective top-gate material (AZO or Ti) and the usage of a HCl dip are indicated. The inset shows a cross-sectional scheme of the geometry with the metallic top gate and the epitaxial n^++^-doped back gate. (**c**) PL spectra from QD1 with AZO top-gate at an excitation power of *P* = 209 nW and varied *U*, as indicated. The exciton(X) peak is marked. For *U* = 0 V, the intensity is multiplied by a factor of 10.

Figure 1a shows a schematic cross-sectional shape of a CSQD, as used in the simulations. This unique shape allows a wide tunability of the charge carrier probability densities by vertical or lateral electric [14] and magnetic [18] fields. Simulations predict, for instance, the transition from a dot to a ring-like hole probability density by a vertical electric field [14].

For the application of a vertical electric field *F*, the CSQDs are integrated into a Schottky diode (inset in Figure 1b). The integrated back gate is realized as a highly n-doped epitaxial layer. In earlier experiments, we used conventional metallic Ti top-gates [14]. To avoid an interface oxide layer, a HCl dip was performed before metal deposition which removes the oxide at the semiconductor surface. For the present experiments, we used a different top-gate material. Here, the usage of Al-doped ZnO (AZO) as an optically transparent and conductive oxide for the top gate is expected to reduce the light absorption. However, the oxygen-based deposition of the AZO layers using atomic layer deposition (ALD) induces an oxidation of the semiconductor surface below the AZO. The ALD process is described detail in in ref. [19]. The AZO top-gate consists of two layers: first a 9 nm thick Al_2_O_3_ layer as insulator, and second, a 21 nm AZO layer as gate electrode. As a reference, data from samples with 10 nm thick Ti top-gates with and without HCL dip are also shown.

Single GaAs CSQDs are studied with micro photoluminescence (PL) using a focused laser with a wavelength of 520 nm for excitation. The experimental conditions are described in ref. [14]. In brief, the samples are installed in a closed-cycle optical cryostat at a temperature of *T* = 4 K. Spectra are taken using an *f* = 750 mm monochromator. For lifetime measurements, the laser is switched to a pulsed mode with 100 ps pulse length and 10 MHz repetition rate. The time evolution is detected using an avalanche photo diode (APD) installed at a *f* = 500 mm monochromator.

The experimental data are compared with the results of a simulation model, which is described in detail in ref. [14]. The finite element model (FEM) in effective mass approximation assumes a rotationally symmetric quasi two-dimensional symmetry and computes the electron and hole eigenenergies and the corresponding wave functions. The Coulomb interaction energy is calculated from the wave functions via the Coulomb integral and used together with the eigenenergies to calculate the energy of the ground-state exciton. In addition, the lifetime in the strong confinement regime is calculated from the overlap integral of the electron and hole wave functions. This topic is addressed in detail in Section 3.3.

## 3. Results

In the following, results from five different QDs are discussed, named QD1, …, QD5. All QD samples are from the same MBE-grown wafer, however, with two different top-gate materials (AZO or Ti with and without HCl dip).

### 3.1. Gate Material

The current density *I* between the metallic top gate and the epitaxial n^++^-doped back gate as a function of the gate voltage *U* is measured to evaluate the different gate materials and the usage of an HCl dip (Figure 1b). In agreement with the usual behavior of a Schottky diode, there is an abruptly increasing *I* in forward bias above a threshold voltage $U_{S}$, which is related to the height of the respective Schottky barrier. The lowest threshold voltage is observed for the AZO gate, where a significant *I* = 3 µA µm^−2^ flows already at *U* = 0.94 V. This means the Schottky barrier height of the AZO gate is lower compared with the Ti gates, where a current density of *I* = 3 µA µm^−2^ flows at *U* = 1.53 V (no HCl dip) and 1.60 V (with HCl dip). The lower Schottky barrier height of AZO can be explained by a lower work function or a higher electron affinity. Furthermore, the slope of the I(U) curves for Ti is influenced by the usage of an HCl dip, which can be caused by an additional oxide layer at the interface to the top gate without an HCl dip. Measurements under a high current can be problematic, since the additional fluctuating charges in the surrounding of the QDs can modify the QD energetic states and broaden the optical linewidth. In addition, the induced electrical power can yield an increasing sample temperature. As a consequence, measurements in the forward bias regime of a Schottky diode must be interpreted with care.

Figure 1c shows typical PL spectra from QD1 at varied gate voltage *U* in the reverse bias regime of the top-gate Schottky diode. The dominant exciton peak (X) is identified from PL measurements with varied excitation power *P*. A comparison of PL data from several QDs with either AZO or Ti with HCl dip as top-gate material demonstrates for the AZO gates on average a 3.3 times higher intensity of the exciton emission for $U≃U_{S}$. For this comparison, the power of the exciting laser is adjusted such that the intensities of the exciton and biexciton peaks are equal. This means the excitation power at the QD position is almost constant and the higher measured exciton intensity for the AZO top-gates is related to the reduced absorption of the light emitted by the QDs.

### 3.2. Stark Shift

The gate-voltage-dependent PL spectra in Figure 1c clearly indicate a *U*-dependent shift of the exciton energy $E_{X}$, which is well known as quantum-confined Stark effect [20,21,22,23,24,25]. Furthermore, starting from $U_{0}≃$ 1.0 V and going down to *U* = 0, we note a reduction in the intensity and also a broadening of the peak for U≤ 0.4 V. For an evaluation of the data in terms of the vertical electric field, the applied gate voltage *U* must be converted into the field strength *F*. In a parallel-plate capacitor model, the relation between *U* and the resulting electric field is $F=-(U-U_{0})/d$, with the distance *d* = 200 nm between the top and the back gate. The value of $U_{0}$ reflects the built-in potential of the top-gate Schottky contact plus the zero-field polarization of the charge carriers (the determination of $U_{0}$ is described below). This means the intensity of the exciton peak is reduced with increasing *F*, which is probably caused by a field-induced escape of charge carriers from QD confinement [25]. The broadening of the exciton peak at higher *F* can be caused, e.g., by fluctuating charges in the surrounding of the QD [26]. Here, fluctuating charges mean traps, where the time-dependent population can be modified by an increasing *F*. As an additional explanation, the assumed weak confinement regime for the hole at increasing *F* (see below) can be slightly nonuniform, which causes variations in the emission energy. In a strong confinement, the charge carriers are localized, and their energy can be calculated using equilibrium approaches. Instead, in a weak confinement, the charge carriers oscillate and experience the local nonuniformities of the confinement.

**Figure 2:**
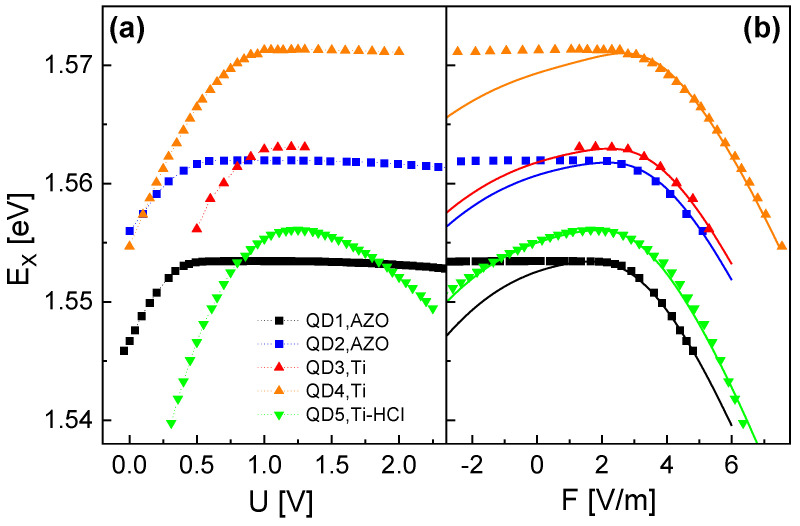
(**a**) Measured exciton energy $E_{X}$ as function of *U* for several QDs. The respective top-gate material (AZO or Ti) and the usage of a HCl dip are indicated. (**b**) Comparison of measured $E_{X}$ (symbols) with simulation results (lines) as function of $F=-(U-U_{0})/d$. In the simulations, the value of $U_{0}$ as well as the QD size-related parameters $r_{QD}$, $d_{QD}$, and $h_{QD}$ are determined by a comparison with the experimental data for $U\leq U_{0}$. The resulting values are given in Table 1.

The measured exciton Stark shift $E_{X}\left(U\right)$ for the discussed QDs is summarized in Figure 2a. In general, the data show an increasing $E_{X}$ with increasing *U* up to a maximum at $U_{max}$. For $U>U_{max}$, the exciton energy either saturates or decreases. A decrease in $E_{X}$ is observed for QD5 (Ti gate with HCl dip) and is in agreement with the often observed parabolic Stark shift [22,23,24,25]. The asymmetry is related to the asymmetric shape of the cone-shell QDs along field direction [14]. On the other hand, the saturation of $E_{X}$ for QD1, …, QD4 can be caused by the additional oxide layer between the AlGaAs barrier and the metallic top gate. A possible scenario is a screening of the voltage-induced electric field by charges inside the oxide layer that are generated at voltages exceeding $U_{max}$. For QD5, the HCl dip removed the oxide layer.

The variation in the maximum $E_{X,max}$ = 1.5534 …1.5713 eV is originated by fluctuations in QD size. All QD samples are from the same wafer and should be nominally equal. However, due to local fluctuations in the process conditions, the studied QDs are not perfectly identical, which yields the observed variation in $E_{X}$. The standard deviation of the measured $E_{X}$ values is 7.0 meV, which is quite narrow compared with other QD systems.

In the next step, we compare the experimental values of $E_{X}$ with results of the FEM simulation. For a relation between the experimental *U*-dependent data and the simulated *F* dependence, the above parallel-plate capacitor model is used. Here, $U_{0}$ is an open parameter. Further open parameters are related to the QD size. We consider a QD shape as illustrated in Figure 1a, with the characteristic lengths $r_{QD}$, $d_{QD}$, and $h_{QD}$. The value of $U_{0}$ and the QD size-related parameters are determined by a comparison between the measured and the simulated Stark shift for $U<U_{0}$. Figure 2b demonstrates the very good reproduction of the measured $E_{X}$ by the simulation. For QD5, with Ti gate and HCl dip, the agreement is almost perfect over the whole field range. For the QDs with AZO gate or Ti gate without HCl dip, the agreement is very good only for $U<U_{0}$; the deviation for higher *U* is caused by the additional oxide layer, as described above.

Table 1 shows the values of the fit parameters as determined by the comparison between the measured and simulated $E_{X}\left(U\right)$. The values of $U_{0}$ are close to the measured threshold voltage $U_{S}$, at which a significant current starts to flow through the Schottky diode (see Section 3.1). Again, the AZO gates show the lowest $U_{0}$ and the Ti gates much higher values. The determined QD sizes, and in particular the value of $h_{QD}$, directly scale with the reciprocal of the maximum exciton energy $U_{X,max}$. This trend agrees with the well-known size quantization effect.

### 3.3. Lifetime

**Figure 3:**
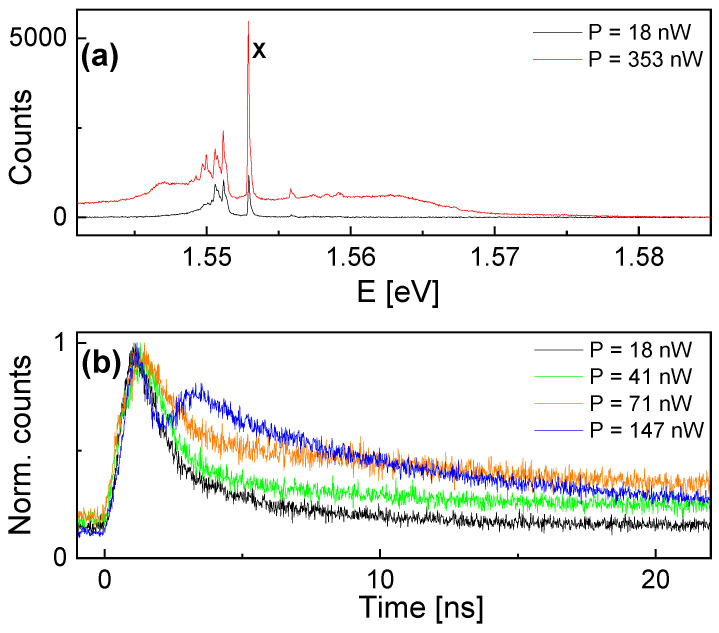
(**a**) PL spectra from QD1 taken using a pulsed laser at excitation powers *P* = 18 nW and 353 nW. The exciton (X) peak is marked. (**b**) Normalized time-dependent exciton peak intensity at varied *P*. All spectra are taken at *U* = 0.9 V.

Time-dependent PL measurements of the exciton peak intensity are taken using a pulsed laser for excitation with a 100 ps pulse length and a repetition rate of 10 MHz. As an example, Figure 3 demonstrates the crucial influence of the excitation power *P* on the emission of QD1. At a very high *P* = 353 nW, there is a significant background in the PL spectrum, which is not visible at a low *P* = 18 nW (Figure 3a). Accordingly, the time-dependent exciton intensity shows at *P* = 353 nW and even two peaks at 147 nW, where the second one is attributed to the background (Figure 3b). This demonstrates that lifetime measurements require a careful choice of excitation power. We attribute the artifacts at higher *P* to the population of excited states at high energy, for instance, in the QD p-shell. These states act as a reservoir and refill the ground state after the radiative recombination of ground-state excitons. This means the second peak in the time-dependent intensity is related to the excited-state lifetime and the time scale for the relaxation of excitons from an excited state to the ground state. Importantly, the excited states are not populated at a low excitation power, as is performed in the present experiments for the characterization of the ground-state excitons. Accordingly, the spectra at P≤ 41 nW demonstrate neither a background (Figure 3a) which can be related to emission from exited states nor a second peak in the lifetime data (Figure 3b). The analyzed lifetimes show a decrease with decreasing *P* and saturate for P≤ 41 nW. This clearly confirms the reliability of lifetime data taken at low P≤ 41 nW. Accordingly, QD1 and QD2 are measured at *P* = 18 nW, and QD3 and QD4 at *P* = 54 nW (there, a background becomes visible at higher P≥ 120 nW). The lower possible excitation power of QD1 and QD2 is a benefit of the optically transparent AZO gates.

For the determination of the lifetime, the measured time-dependent exciton intensity is fitted using a biexponential decay:

(1) $$I\left(t\right)=A_{F}\exp\left(-t/\tau_{F}\right)+A_{S}\exp\left(-t/\tau_{S}\right)+I_{0}$$

with constants $A_{F}$, $A_{S}$, $I_{0}$, and the fast $\tau_{F}$ and slow $\tau_{S}$ decay times. Such determined values are interpreted based on a rate model of radiative (bright) and nonradiative (dark, the charge carrier spin orientation does not allow photon emission) recombination processes [17], which yields, for the bright and dark recombination rates,

(2) $$R_{B}=+\frac{A_{F}}{A_{F}+A_{S}}\tau_{F}^{-1}+\frac{A_{S}}{A_{F}+A_{S}}\tau_{S}^{-1}$$

(3) $$R_{D}=-\frac{A_{S}}{A_{F}-A_{S}}\tau_{F}^{-1}+\frac{A_{F}}{A_{F}-A_{S}}\tau_{S}^{-1}$$

where $\tau_{B}=1/R_{B}$ and $\tau_{D}=1/R_{D}$ are the corresponding bright and dark lifetimes.

**Figure 4:**
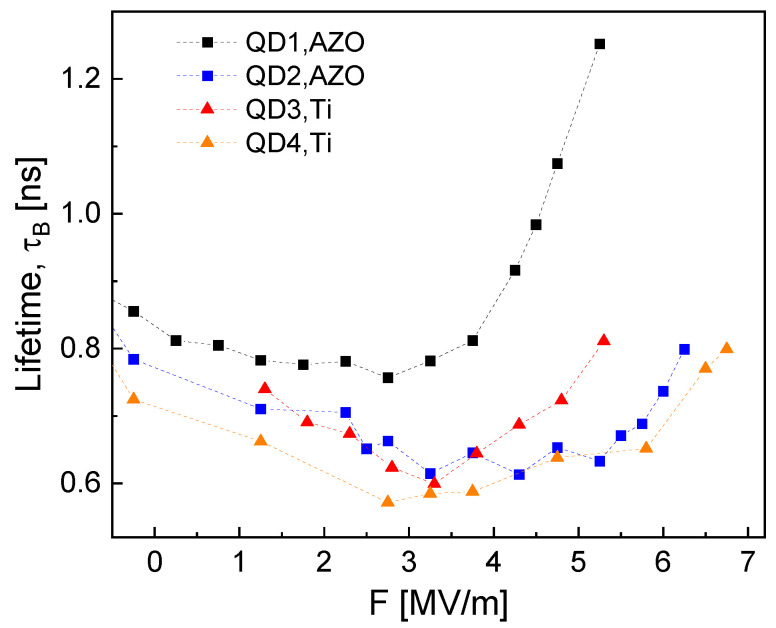
Measured bright lifetime $\tau_{B}$ as function of *F* for several QDs.

Figure 4 shows a comparison of measured values of $\tau_{B}$ as function of *F* for QD1, …, QD4. For all dots, the trend is quite similar, with a minimal $\tau_{B}$ = 0.58 …0.77 ns at about *F* = 3 MV/m and an increase with *F*. In agreement with the influence of the QD size in ref. [17], larger QDs with a smaller $E_{X}$ have a longer $\tau_{X}$ (Table 1).

In the following, lifetime data from QD1 are discussed, since QD1 with the largest size allows the widest variation in the lifetimes via an external electric field. From the measured $\tau_{B}$, the experimental oscillator strength $f_{PL}$ is calculated using [1]

(4) $$f_{PL}=3h^{2}c^{3}\epsilon_{0}m_{0}/\left(2n\pi q^{2}E_{X}^{2}\tau_{B}\right)$$

with Planck’s constant *h*, the speed *c* of light, the vacuum permittivity $\epsilon_{0}$, the electron mass $m_{0}$, the refractive index *n* of the AlGaAs barrier, the elementary charge *q*, and the exciton energy $E_{X}$.

In the simulations, as a first approach, the oscillator strength is calculated for a homogeneous medium from the overlap integral ${\langle \psi_{e}|\psi_{h}\rangle }^{2}$ between the electron and hole envelope wave functions in the QD [4]

(5) $$f_{S}=E_{P}{\langle \psi_{e}|\psi_{h}\rangle }^{2}/E_{X}$$

with the Kane energy $E_{P}$. This approach is related to the so-called strong confinement regime [1,4], where the QD radius $r_{QD}$ is smaller than the bulk exciton Bohr radius $\lambda_{B}=4\pi \epsilon_{s}\epsilon_{0}\hbar^{2}/\left(m_{x}^{*}q^{2}\right)$, with the semiconductor dielectric constant $\epsilon_{s}$, the exciton effective mass $m_{x}^{*}={\left(1/m_{e}^{*}+1/m_{hh}^{*}\right)}^{-1}$, the electron $m_{e}^{*}$, and the heavy hole effective mass $m_{hh}^{*}$. For GaAs, $\lambda_{B}$ = 11.7 nm. In a strong confinement, the maximal oscillator strength for ${\langle \psi_{e}|\psi_{h}\rangle }^{2}=1$ is $f_{max}=E_{P}/\left(\hbar \omega_{0}\right)$ = 18.5, with $E_{X}$ = 1.562 eV and $E_{P}$ = 28.8 eV. Now, the radiative lifetime $\tau_{S}$ of an exciton in a strong confinement can be calculated from the simulated wave functions by combining Equations (4) and (5)

(6) $$\tau_{B}=3h^{2}c^{3}\epsilon_{0}m_{0}/\left(2n\pi q^{2}E_{X}^{2}f_{S}\right)$$

**Figure 5:**
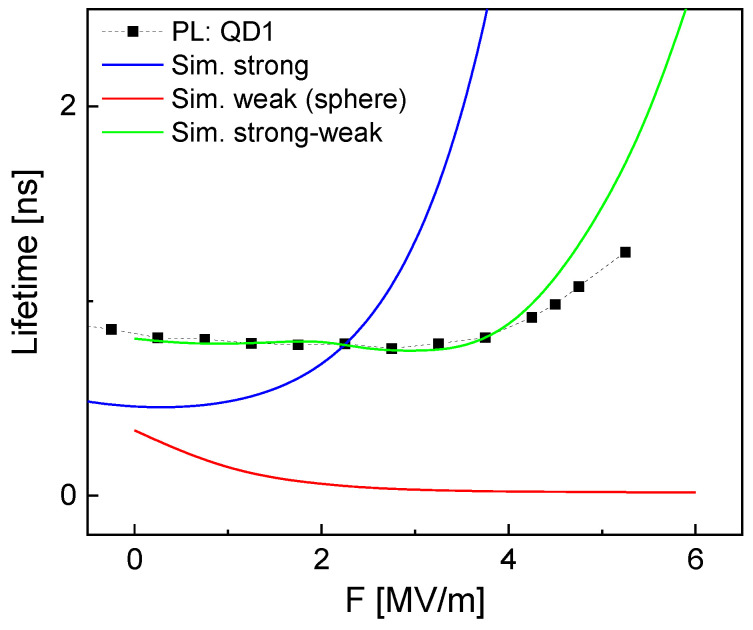
Comparison of measured and simulated lifetimes of QD1. Different simulation models are used assuming the strong confinement regime (Sim. strong), the weak confinement regime for a spherical QD (Sim. weak), and an empirical approach combining strong and weak confinement (Sim. strong–weak).

Figure 5 compares measured and simulated lifetimes of QD1. For simulation results interpreted assuming a strong confinement (Equation (6)), there are fundamental disagreements between experimental and simulated lifetimes, in particular at higher *F*. For instance, at *F* = 5 MV/m, the simulated $\tau_{S}$ = 7.2 ns is more than six times longer than the measured $\tau_{PL}$ = 1.15 ns.

**Figure 6:**
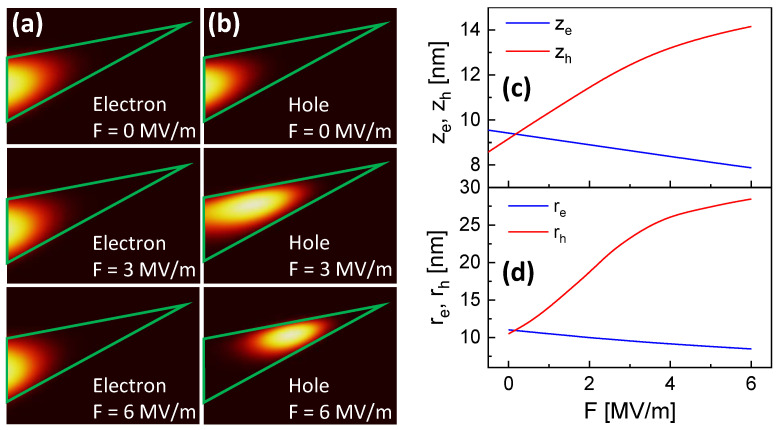
(**a**) 35 × 25 nm color-coded cross sections through simulated electron probability densities at varied *F*. The green lines indicate the shape of QD1. (**b**) Cross-sections through simulated hole probability densities at varied *F*. (**c**) z-position of the electron $z_{e}$ and hole $z_{h}$ probability density barycenters as function of *F*. (**d**) Radius of the electron $r_{e}$ and hole $r_{h}$ probability densities as function of *F*. For the radius, the point of the reduction in the probability density to 1/e is taken.

We assume that the deviation between experiments and simulations is caused by the assumption of a strong confinement, which is not applicable to the present CSQDs under a vertical electric field. This is illustrated by the simulated probability densities shown in Figure 6a,b. An increasing *F* pushes the hole into the wing part of a CSQD, where it forms a quantum ring with an increased effective radius. In contrast to the hole, an increasing *F* pushes the electron into the CSQD tip, and it remains small. Figure 6c shows the simulated z positions of the electron and hole barycenters and Figure 6d the corresponding radii of the electron and hole probability densities. As an approximation for the radii $r_{e}$ and $r_{h}$, we take the point at which the corresponding probability density is reduced to 1/e. With $r_{e}$ and $r_{h}$ as effective quantum dot radii, the electron radius is always smaller than $\lambda_{B}$ and, thus, the electron remains in the strong confinement regime. On the other hand, at F= 0.4 MV/m, the hole radius $r_{h}$ starts to exceed $\lambda_{B}$, and the hole switches from a strong to weak confinement.

In weak confinement, a so-called giant oscillator strength is assumed [13] with much higher values compared with strong confinement. Stobbe et al. [13] calculated the oscillator strength of a sphere in a weak confinement as

(7) $$f_{W,sphere}=f_{max}\sqrt{\pi }{\left(r_{QD}/\lambda_{B}\right)}^{3}$$

Interestingly, now *f* depends on the ratio $r_{QD}/\lambda_{B}$ and not on ${\langle \psi_{e}|\psi_{h}\rangle }^{2}$. When we replace $r_{QD}$ in Equation (7) by $r_{h}$, we obtain a very large oscillator strength with values ranging from 24 at *F* = 0 up to 477 at *F* = 6 MV/m. The corresponding lifetimes are calculated using Equation (6), with $f_{S}$ replaced by $f_{W,sphere}$, and are plotted in Figure 5. Obviously, now the simulated lifetimes are unrealistically short compared with the experiments, which is caused by the giant $f_{W,sphere}$ in the sphere approximation.

A more realistic oscillator strength for the present CSQDs should consider the strong confinement of the electron and the weak confinement of the hole in the form

(8) $$f_{SW}=f_{S}\left({\langle \psi_{e}|\psi_{h}\rangle }^{2}\right)f_{W}\left(r_{h}/\lambda_{B}\right)$$

**Figure 7:**
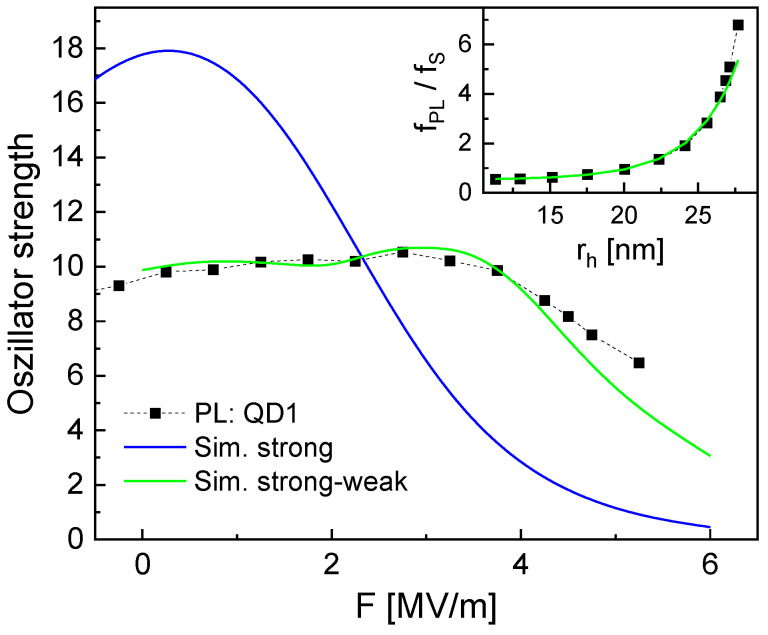
Comparison of measured and simulated oscillator strength *f* of QD1. Different simulation models are used assuming the strong confinement regime (Sim. strong) and an empirical approach combining strong and weak confinement (Sim. strong–weak). The inset shows $f_{W}=f_{PL}/f_{S}$ as function of $r_{H}$.

Here, the experimental $f_{PL}$ calculated using Equation (4) is equivalent to the simulated $f_{SW}$. Figure 7 compares the experimental $f_{PL}$ and the simulated $f_{S}$ for strong confinement. To illustrate the trend of $f_{W}\left(r_{h}/\lambda_{B}\right)$, the ratio $f_{PL}/f_{S}$ is plotted as function of $r_{h}$ in the inset of Figure 7). A fit yields an expression $f_{W}=0.50+0.063{\left(r_{h}/\lambda_{B}\right)}^{2.13(r_{h}/\lambda_{B})}$. However, we note that this is only an empirical expression without a physical model behind it. The simulated $f_{SW}$ calculated in this way shows a good agreement with the experimental $f_{PL}$ (Figure 7).

Now, the lifetime in the combined strong–weak confinement is calculated using Equation (6) with $f_{S}$ replaced by $f_{SW}$ and is plotted in Figure 5. Again, the simulations assuming an asymmetric strong–weak confinement demonstrate a good reproduction of the experimental data. This supports the present assumption of an *F*-induced confinement switching for the hole.

## 4. Discussion and Conclusions

AZO as top-gate material demonstrates a more than three times higher intensity of the exciton peak in comparison with the often-used Ti gates. This advantage is related to the reduced optical absorption. On the other hand, the preparation of the AZO gates causes an interface oxide layer that yields artifacts for gate voltages above U≃ 1.0 V. This limits reliable measurements to the range of positive electrical fields.

The main part of this study addresses the optical properties of GaAs cone shell QDs in a vertical electric field. As the central finding, we observe a substantial discrepancy at high electric fields between the measured bright lifetime and simulation results, assuming the usual strong confinement regime. Here, the simulation computes much longer lifetimes compared with the experiments. Since the simulation reproduces the experimental Stark shift data very well, we consider the simulation model reliable and attribute this discrepancy to the assumption of a strong confinement regime. Further simulations predict the *F*-induced transformation of the shape of the hole probability density from a dot into a ring. This shape transformation is possible due to the unique shape of the CSQDs. In the ring state, the effective radius of the hole confining potential becomes larger than the bulk exciton Bohr radius $\lambda_{B}$. Now, the assumption of a strong confinement for the hole is not further valid, and we have to consider a weak confinement. On the other hand, since *F* pushes the electron into the tip part of a CSQD, its shape always remains like a dot in a strong confinement. This means that at a large *F*, we have an interesting asymmetric confinement with a weakly confined hole and a strongly confined electron. An empirical approach for the oscillator strength in this asymmetric strong–weak confinement demonstrates a good agreement between the experimental and simulated bright lifetimes. However, a deeper theoretical study of this asymmetric confinement is desirable.

So far, the *F*-induced transformation of the hole probability density in a CSQD from a dot into a quantum ring was a theoretical prediction [14]. Now, the present study supports this prediction by indicating that the assumption of a strong confinement regime in the simulation yields only a poor reproduction of the experimental lifetime data. A weak confinement of the hole, which is compatible with the expected ring formation, is demonstrated as a reasonable explanation. Quantum rings are highly interesting objects of the nanoworld [27], and for CSQDs in combined electric and magnetic fields, application as storage for photo-excited charge carriers is predicted [18].

## Figures and Tables

**Table 1:** Comparison of the discussed QDs with varied gate material (AZO or Ti) and with or without HCl dip. The values of $U_{0}$, $r_{QD}$, $d_{QD}$, and $h_{QD}$ are determined by a comparison between experimental and simulated $E_{X}\left(U\right)$, as shown in Figure 2b.

Dot	Gate Material	HCl Dip	$E_{X,\max}$ [eV]	$U_{0}$ [V]	$r_{\mathrm{QD}}$ [nm]	$d_{\mathrm{QD}}$ [nm]	$h_{\mathrm{QD}}$ [nm]	$\tau_{X}$ (*F* = 3 MV/m) [ns]
QD1	AZO	no	1.5534	0.92	35.0	19.0	13.5	0.77
QD2	AZO	no	1.5619	1.02	33.0	18.0	11.9	0.63
QD3	Ti	no	1.5631	1.56	33.0	18.0	11.7	0.61
QD4	Ti	no	1.5713	1.51	33.0	18.0	10.5	0.58
QD5	Ti	yes	1.5560	1.58	35.0	19.0	12.8	–

## Data Availability

The data presented in this study are available on request from the corresponding author.
